# Biological Mechanisms of Aflatoxin B_1_-Induced Bile Metabolism Abnormalities in Ducklings

**DOI:** 10.3390/ani14202996

**Published:** 2024-10-17

**Authors:** Yihong Chu, Aimei Yu, Huanbin Wang, Shahid Ali Rajput, Qianqian Yu, Desheng Qi

**Affiliations:** 1 Department of Animal Nutrition and Feed Science, College of Animal Science and Technology, Huazhong Agricultural University, Wuhan 430070, China; cyhong@webmail.hzau.edu.cn (Y.C.); yam@webmail.hzau.edu.cn (A.Y.); whbin@webmail.hzau.edu.cn (H.W.); yuqianqian@webmail.hzau.edu.cn (Q.Y.); 2 Faculty of Veterinary and Animal Science, Muhammad Nawaz Shareef University of Agriculture, Multan 60000, Pakistan; shahid.ali@mnsuam.edu.pk

**Keywords:** aflatoxin B_1_, duckling, bile metabolism, transcriptome sequencing, liver injury, antioxidant capacity

## Abstract

**Simple Summary:**

Aflatoxin B_1_ is highly toxic and widely prevalent, posing a serious threat to both human and livestock health. In production, it has been observed that aflatoxin B_1_-contaminated feed can cause the liver of animals to turn green, likely due to abnormal bile metabolism. This study investigated the mechanisms of the effect of aflatoxin B_1_ on bile metabolism in ducklings, intending to provide insights into the prevention and control of aflatoxin B_1_ in agricultural practices.

**Abstract:**

This study investigated the effects and biological mechanisms of aflatoxin B_1_ (AFB_1_) on the health and bile metabolism of ducklings. Forty-eight 1-day-old ducklings were randomly assigned to two groups, with six replicates per group. The control group was fed a basic diet, while the AFB_1_ group received a diet containing 90 µg/kg of AFB_1_. The experiment lasted for 2 weeks. The results showed that 90 µg/kg AFB_1_ caused abnormal bile metabolism; damaged liver cell nuclei and mitochondria; and significantly decreased body weight, average daily weight gain, and levels of albumin, total protein, cholesterol, total superoxide dismutase, glutathione peroxidase, and glutathione. It also significantly increased feed conversion efficiency, along with alanine aminotransferase, aspartate aminotransferase, alkaline phosphatase, total bile acids, and malondialdehyde levels. In the liver, the expression levels of *CYP7A1*, *SCD*, and other genes were significantly upregulated, while *BSEP*, *FASN*, *HMGCR*, *CAT*, and other genes were significantly downregulated. In conclusion, AFB_1_ causes abnormal bile metabolism and impairs the overall health and liver function of ducklings. Its mechanism of action may involve changes in gene expression related to bile acid metabolism, lipid metabolism, oxidative damage, and cancer pathways.

## 1. Introduction

Aflatoxins are a type of mycotoxin commonly found in agricultural production that pose a significant threat to both food safety and animal health [1,2]. When ingested by animals, AFB_1_ can cause both acute and chronic poisoning, leading to serious health issues [2]. Sensitivity to AFB_1_ varies across species, with ducklings being among the most susceptible [3,4]. During production, it was observed that ducks’ livers turned green when feed exceeded the AFB_1_ safety threshold (10 µg/kg: Chinese Feed Hygiene Standards/GB 13078-2017), suggesting abnormalities in bile metabolism [5]. Such abnormalities may exacerbate liver damage and influence gene expression in various hepatic metabolic pathways [5,6]. At the same time, previous research in our laboratory has also found that AFB_1_-contaminated feed can cause bile stasis in ducks at 28 days. A preliminary study has been conducted to explore the possible reasons for this, which may be related to changes in the expression levels of bile acid synthesis and excretion genes in the liver [7]. However, the timing of the initial occurrence of bile stasis in ducks and changes in the transcriptome response of the liver after bile stasis are still unclear.

The liver plays a crucial role in metabolism and detoxification in animals, and liver damage is often the most evident consequence of AFB_1_ poisoning [3,8]. In the liver, AFB_1_ undergoes two main processes: activation and detoxification [3,9]. The metabolic activation of AFB_1_ involves the CYP450 enzyme system, which also produces large amounts of reactive oxygen species (ROS) [3,10]. The accumulation of ROS can damage mitochondria and trigger apoptosis in liver cells [10,11]. Furthermore, ROS build-up can lead to oxidative stress in the liver, increasing malondialdehyde (MDA) levels [10]. During AFB_1_ metabolism, the metabolite AFB_1_-exo-8,9-epoxide binds with glutathione (GSH) and is subsequently excreted from the body [3,9]. As a result, AFB_1_ poisoning depletes GSH and reduces the activity of antioxidant enzymes such as catalase (CAT), superoxide dismutase (SOD), and glutathione peroxidase (GPX) while simultaneously increasing the levels of peroxides such as MDA [12].

Abnormal bile metabolism can further aggravate liver damage [5,6]. Bile acids, synthesized from cholesterol in the liver, are the main component of bile [13]. The gene *CYP7A1* plays a key role in regulating bile acid synthesis, while *HMGCS1* and *HMGCR* regulate cholesterol synthesis [13,14,15,16]. Altered expression of these genes can impact bile acid production [13,14,15,16]. Once synthesized, bile acids are secreted by the liver into the bile duct and stored in the gallbladder [13,17]. When released into the duodenum, bile aids fat digestion and absorption [17]. Bile secretion is regulated by the gene encoding the bile salt export pump (*BSEP*), facilitating the transport of bile acids from liver cells into the bile duct [17,18]. Abnormalities in these processes can lead to abnormal bile metabolism, further impairing liver function.

In order to determine the earliest time point when AFB_1_ causes bile stasis in duck liver and study the changes in gene expression levels in various metabolic pathways of the liver after bile stasis occurs, this study utilized 48 1-day-old Cherry Valley ducks. After observing the effects of AFB_1_ on the overall health and liver function of the ducklings, particularly noting abnormal bile metabolism in the second week, transcriptome sequencing was performed to detect changes in hepatic transcriptome responses. Quantitative polymerase chain reaction (qPCR) was also used to validate the transcriptomic findings. This study provides valuable insights into AFB_1_ poisoning and its link to bile metabolism abnormalities.

## 2. Materials and Methods

### 2.1. Ducklings, Treatments, and Sample Acquisition

This study was approved by the Science Ethics Review Committee of Huazhong Agricultural University (approval number HZAUDU-2024-0008). One-day-old Cherry Valley meat ducklings were purchased from Wuhan Yongsheng Duck Industry Co., Ltd. (Wuhan, China). The experiment consisted of a control group and an AFB_1_ treatment group, with six replicates in each group and four ducklings in each replicate. The AFB_1_ used in the study was derived from laboratory-cultured fermented products. The basic dietary composition and nutritional levels are provided in Table 1. The diet of the AFB_1_ group was supplemented with 90 µg/kg of AFB_1_. (The actual content of AFB_1_ in the diet was determined using a commercial reagent kit (Romer Labs, Getzersdorf, Austria) [7], and the actual content of AFB_1_ was 93 µg/kg.) The ducklings were housed in stainless steel cages with free access to feed and water. Before the formal experiment began, the floor, water dispenser, feeding trough, and duck cage of the duck house were thoroughly cleaned. After the duck house dried, it was sealed and fumigated with formalin and potassium permanganate for disinfection. The doors and windows were opened for three days for ventilation. When the ducklings were 1–3 days old, the heater of the duck house was turned on every day to ensure the indoor temperature remained between 30 and 34 °C. After 4 days of age, the indoor temperature was gradually decreased. The experiment lasted for 2 weeks, and the production performance of the ducklings was recorded weekly.

The ducklings were weighed at the start of the experiment and again at the end of the first and second weeks. At the conclusion of each week, one duckling from each replicate was randomly selected for blood collection from the wing vein. The serum was isolated and stored at −80 °C for subsequent indicator analyses. After using ether to make all the ducklings unconscious, the ducklings were euthanized by acute bloodletting. The ducklings were slaughtered and sampled. The heart and liver were weighed, and a photograph of the liver was taken. Two small pieces of liver tissue were fixed in 2.5% glutaraldehyde and 4% paraformaldehyde solutions, respectively, to prepare ultra-thin paraffin sections. The remaining liver tissue was stored at −80 °C for further indicator analyses.

### 2.2. Performance Testing

The initial weight of the ducklings was recorded prior to the start of the experiment. The ducklings were then weighed at the end of the first and second weeks to calculate the average daily weight gain. The daily feed intake was recorded, and the remaining feed in the feeders was weighed at the end of each week to calculate the average daily feed consumption. The feed conversion rate was calculated based on the total feed consumed and the weight gain achieved.

### 2.3. Determination of Serum Biochemical Indicators

The levels of alanine aminotransferase (ALT), aspartate aminotransferase (AST), alkaline phosphatase (ALP), total protein (TP), albumin (ALB), triglycerides, cholesterol, total bile acids (TBA), total bilirubin, and direct bilirubin in the serum were measured using a fully automated biochemical analyzer (Beckman, Brea, CA, USA).

### 2.4. Determination of Bile Acid and Antioxidant Enzyme Content in Liver

The contents of TBA, total SOD (T-SOD), MDA, GPX, and GSH in the liver were determined using a commercial kit (Nanjing Jiancheng Biotechnology Co., Ltd., Nanjing, China), following the manufacturer’s instructions for each assay.

### 2.5. Haematoxylin and Eosin (H&E) Staining Method

The liver tissue blocks were first immersed in a 4% paraformaldehyde solution for 48 h to ensure complete fixation. The tissue blocks were then removed and dehydrated using graded ethanol solutions. Once dehydrated, the tissue blocks were embedded in paraffin using an embedding machine to create wax blocks. A surgical knife was used to smooth the wax blocks, which were then sectioned into slices approximately 3 µm thick using a paraffin microtome. The sections were mounted onto glass slides, dried, and stained with H&E. Following ethanol dehydration, the slides were mounted with a coverslip and sealed with neutral gum for preservation. The sections were observed and photographed using a microscope (Olympus Co., Ltd., Tokyo, Japan).

### 2.6. Oil Red O Staining Method

The liver tissue blocks were fixed in a 4% paraformaldehyde solution. This was followed by sequential dehydration in 15% and 30% sucrose solutions. The tissue blocks were then embedded in an optimal cutting temperature compound and sectioned into slices approximately 8 µm thick using a cryostat. The sections were mounted onto glass slides, dried, and fixed with a fixative. The slides were immersed in Oil Red O staining solution for 8 min, followed by haematoxylin staining for 3 min. After staining, the sections were covered with a coverslip and sealed with glycerol gelatine. The slides were then observed and photographed under a microscope (Olympus Co., Ltd., Tokyo, Japan).

### 2.7. Observation and Analysis of Liver Ultrastructure

The liver tissue blocks were fixed in a 2.5% glutaraldehyde solution for 24 h. This was followed by three washes with phosphate-buffered saline. The tissue blocks were then fixed in osmium tetroxide solution for 2 h and washed thrice with phosphate-buffered saline. After dehydration with acetone, the tissue blocks were embedded in resin. Ultra-thin sections, approximately 60 nm thick, were prepared from the embedded tissue blocks. The sections were mounted onto copper grids and stained with uranyl acetate solution. The sections were then observed and photographed using a transmission electron microscope (Hitachi H-7650, Tokyo, Japan).

### 2.8. Liver Transcriptome Sequencing

RNA sequencing analysis was conducted on liver samples from both the control and AFB_1_ groups, with six replicates in each group. Total RNA was extracted from the liver using Trizol (ABclonal Technology Co., Ltd., Wuhan, China), and mRNA was enriched using magnetic beads conjugated with Oligo (dT). Following reverse transcription to synthesize cDNA from the mRNA template, double-stranded cDNA was synthesized, purified, and repaired. PCR technology was then employed to enrich the cDNA and construct a cDNA library (Luo et al., 2019) [19]. After constructing the cDNA library, sequencing analysis was performed using an Illumina NextSeq 500 sequencer (Illumina, San Diego, CA, USA).

The sequencing results were aligned with the reference genome *Anas platyrhynchos* (https://www.ncbi.nlm.nih.gov/data-hub/genome/GCA_000355885.1/ accessed on 31 May 2024), using version GCF000355885.1 of the reference genome. Genes with a fold change in expression of ≥1.2 and *p* < 0.05 between the AFB_1_ and control groups were defined as differentially expressed genes. Gene Ontology (GO) and Kyoto Encyclopedia of Genes and Genomes (KEGG) enrichment analyses were performed on these differentially expressed genes.

### 2.9. Real-Time qPCR

To validate the accuracy of the transcriptome sequencing results, eight genes were randomly selected for real-time qPCR analysis: cholesterol 7-alpha-monooxygenase (*CYP7A1*), cholesterol 25-hydroxylase (*CH25H*), cholesterol 24-hydroxylase (*CH24H*), solute carrier family 51 beta subunit (*SLC51B*), stearoyl-CoA desaturase (*SCD*), stearoyl-CoA desaturase 5 (*SCD5*), superoxide dismutase 1 (*SOD1*), and catalase (*CAT*). The primer sequences are listed in Table 2. The method for extracting total RNA from the liver was the same as described in the transcriptome sequencing section.

Reverse transcription synthesis of cDNA and qPCR experiments were performed according to the method reported by Wang et al. [20]. The *GAPDH* gene was used as the internal reference, and the relative expression levels of each gene were calculated using the 2^−ΔΔCt^ method. During the experiment, different processing steps were divided into different experimental areas for operation, and disposable gloves, suction heads, and centrifuge tubes were used to avoid cross-contamination of the samples.

### 2.10. Statistical Analysis

The experimental results were analyzed using IBM SPSS Statistics 25 software. After conducting a normal distribution test, all experimental results were analyzed using one-way ANOVA and the Tukey test for differences between groups, and the data are presented as mean ± standard deviation. A *p*-value of <0.05 was considered indicative of a statistically significant difference between the two groups.

## 3. Results

### 3.1. Changes in Growth Performance and Organ Weight

The effects of AFB_1_ on the growth performance and organ weight of ducklings are presented in Table 3. There was no significant difference in initial body weight between the control and AFB_1_ groups, with both averaging around 50 g (*p* > 0.05) (Table 3). During the first and second weeks, AFB_1_ significantly reduced both the body weight and average daily weight gain of the ducklings, while significantly increasing feed conversion efficiency (*p* < 0.05) (Table 3). AFB_1_ had no significant effect on average daily feed intake, liver weight, or heart weight (*p* > 0.05) (Table 3).

### 3.2. Changes in Serum Biochemical Indicators

The effect of AFB_1_ on serum biochemical indicators in ducklings is shown in Table 4. During both the first and second weeks, AFB_1_ significantly increased the levels of ALT and AST in serum while significantly reducing the levels of TP and ALB (*p* < 0.05) (Table 4). Additionally, in the second week, AFB_1_ significantly increased the serum ALP level while significantly decreasing the triglyceride and cholesterol levels (*p* < 0.05) (Table 4).

Regarding the serum bile acid content, AFB_1_ had no significant effect on TBA or total bilirubin levels in ducklings during the first week (*p* > 0.05) (Table 4). However, in the second week, the serum TBA levels significantly increased in the AFB_1_ group (*p* < 0.05), and although the total bilirubin level showed an upward trend, the difference was not statistically significant (*p* > 0.05) (Table 4). Changes in the direct bilirubin level were not significant during either the first or second week (*p* > 0.05) (Table 4).

### 3.3. Changes in Liver Bile Acid and Antioxidant Enzyme Content

The effects of AFB_1_ on bile acid and antioxidant enzyme content in duckling liver are shown in Table 5. During the first week, there were no significant changes in liver TBA and MDA levels in the AFB_1_ group (*p* > 0.05), whereas T-SOD, GPX, and GSH levels significantly decreased (*p* < 0.05) (Table 5). In the second week, AFB_1_ significantly increased liver TBA and MDA levels while significantly reducing T-SOD, GPX, and GSH levels (*p* < 0.05) (Table 5).

### 3.4. Changes in Liver Health and Liver Tissue Structure

**Figure 1:**
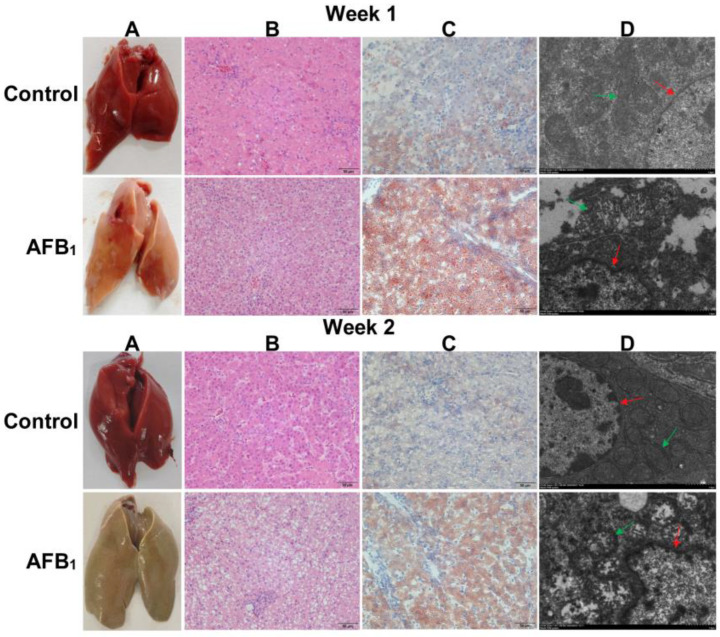
Effect of AFB_1_ on liver health and tissue structure. (**A**) Effect of AFB_1_ on the appearance of the liver. (**B**) H&E staining of liver tissue (200×). (**C**) Oil Red O staining of liver tissue (200×). (**D**) Analysis of the ultrastructure of liver cell nuclei and mitochondria (10,000×). Red arrow: cell nucleus; green arrow: mitochondria.

The effects of AFB_1_ on liver health, tissue structure, and cellular ultrastructure in ducklings are shown in Figure 1. The color of the liver in the control group appeared bright red (Figure 1A). In contrast, the AFB_1_ group exhibited signs of fatty liver during the first week, with the liver appearing yellow (Figure 1A). By the second week, the liver in the AFB_1_ group continued to exhibit signs of fatty liver, as well as signs of abnormal bile metabolism, with the liver turning a yellow-green color (Figure 1A).

H&E staining revealed that the liver cells in the control group were closely arranged, with no signs of steatosis (Figure 1B). In the AFB_1_ group, liver cells displayed vacuolar degeneration caused by fatty infiltration, with increased gaps between the cells (Figure 1B). Oil Red O staining showed a significant accumulation of lipid droplets that were stained orange-red in the AFB_1_ group’s liver (Figure 1C). Ultrastructural analysis of the liver showed that in the control group, the nuclear membrane of liver cells was intact, the mitochondrial cristae were well preserved, and the mitochondrial matrix was densely packed and darker in color (Figure 1D). In contrast, liver cells in the AFB_1_ group displayed nuclear membrane shrinkage, fragmented mitochondrial cristae, and an empty mitochondrial matrix, as observed under transmission electron microscopy (Figure 1D).

### 3.5. Statistical Results of Transcriptome Dataset

The quality of liver transcriptome sequencing is presented in Table 6. In total, 12 samples were used for transcriptome sequencing analysis, and 12 cDNA libraries were constructed. The paired ends yielded between 37,918,248 and 44,565,508 raw reads from the libraries, with Q30 values ranging from 95.42% to 96.29%, all exceeding 80% (Table 6). After quality control, which involved removing low-quality reads, uncertain bases, and adapter sequences, 37,557,418 to 44,055,108 clean reads were obtained (Table 6). When aligned with the *Anas platyrhynchos* genome, the proportion of clean reads mapped to the reference genome ranged from 74.87% to 87.25% (Table 6).

### 3.6. Differential Analysis of Gene Expression Levels

**Figure 2:**
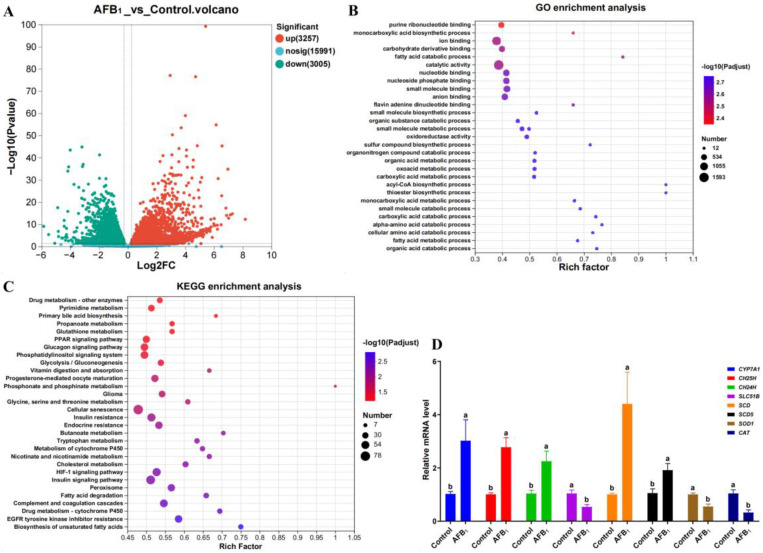
Liver transcriptome sequencing analysis results. (**A**) Differential gene expression status. (**B**) GO enrichment analysis of differentially expressed genes. (**C**) KEGG enrichment analysis of differentially expressed genes. (**D**) qPCR results (*n* = 6), ^a,b^ columns with different owercase letters indicated significant differences between the compared groups (*p* < 0.05).

The liver transcriptome sequencing results are shown in Figure 2 and Supplementary Document S1. In total, 6262 genes were differentially expressed in the liver of the AFB_1_ group, with 3257 genes significantly upregulated and 3005 genes significantly downregulated (Figure 2A) (*p* < 0.05). The GO enrichment analysis revealed that genes with significant differences related to bile acid metabolism and AFB_1_ toxicity were mainly involved in pathways such as oxidoreductase activity, organic acid metabolic processes, fatty acid metabolic processes, and organic acid catabolism (Figure 2B). The KEGG enrichment analysis indicated that genes linked to bile acid metabolism and AFB_1_ toxicity were distributed in pathways such as primary bile acid biosynthesis, GSH metabolism, the PPAR signaling pathway, cellular senescence, cholesterol metabolism, and peroxisomes (Figure 2C).

Table 7 summarizes the differential genes and their expression changes related to bile acid synthesis, bile secretion signaling, lipid synthesis, liver oxidative damage, and cancer pathways. The expression levels of *CYP7A1*, *CH24H*, and *CH25H* in the bile acid synthesis pathway were significantly upregulated (*p* < 0.05), while the expression levels of *BSEP*, *ABCG5*, and *ABCG8* in the bile acid efflux pathway were significantly downregulated (*p* < 0.05). In the lipid synthesis pathway, the expression levels of *SCD* and *SCD5* were significantly upregulated (*p* < 0.05), while *FASN*, *FADS2*, *HMGCS1*, and *HMGCR* were significantly downregulated (*p* < 0.05). Additionally, in the oxidative damage pathway, *CAT*, *SOD1*, *GSTK1*, and *MGST2* expression levels were significantly downregulated (*p* < 0.05).

Real-time qPCR showed that AFB_1_ significantly upregulated the expression of *CYP7A1*, *CH25H*, *CH24H*, *SCD*, and *SCD5* in the liver and significantly downregulated the expression of *SLC51B*, *SOD1*, and *CAT* (Figure 2D) (*p* < 0.05).

## 4. Discussion

### 4.1. The Effect of AFB_1_ on the Production Traits of Ducklings

This study showed that AFB_1_ led to a decrease in body weight and average daily weight gain in ducklings, and increased feed conversion efficiency. Sumantri et al. [21] reported that adding 100 µg/kg AFB_1_ to the diet of laying ducks significantly reduced both body weight and average daily weight gain. Similarly, Wang et al. [22] found that acute toxicity experiments on ducklings with 0.3 mg/kg body weight of AFB_1_ led to reduced body weight and feed intake. Additionally, Feng et al. [23] showed that feeding ducks AFB_1_-contaminated corn significantly reduced their final weight, average daily feed intake, and daily weight gain. In addition, previous research in the laboratory has shown that AFB_1_ also has adverse effects on body weight, daily weight gain, and feed intake of meat ducks at 2–4 weeks [7]. The results of this study align with previous reports, confirming that AFB_1_ poisoning negatively impacts the growth of ducklings.

### 4.2. The Effect of AFB_1_ on the Serum Biochemistry of Ducklings

Serum biochemical indicators reflect the body’s metabolic status [22,24,25]. This study revealed that AFB_1_ significantly increased the levels of three transaminases in the serum of ducklings, suggesting liver damage in the AFB_1_ group [22,24,25]. Simultaneously, the levels of TP and ALB significantly decreased, indicating impaired liver synthesis, a further manifestation of liver injury [22,25]. Additionally, the liver of the AFB_1_ group turned yellow, and large numbers of lipid droplets were observed in the liver tissue, indicating fatty degeneration and abnormal lipid metabolism. Salem et al. [26] found similar effects in broiler chickens, and Altyar et al. [27] showed that the oral administration of AFB_1_ significantly increased ALT, AST, and ALP levels in rats. In addition, laboratory researchers reported that AFB_1_ increased ALT and AST levels in meat duck’s serum while decreasing ALB and TP levels [7,22]. These findings are consistent with previous reports, indicating that AFB_1_ causes liver damage and disrupts body metabolism, which likely contributes to the decline in production performance observed in ducklings after AFB_1_ poisoning.

### 4.3. The Effect of AFB_1_ on Organelle Structure and Antioxidant Indicators in Ducklings

The nucleus and mitochondria are critical organelles in liver cells [28]. In this study, the nuclei and mitochondria of liver cells in the AFB_1_ group were damaged. This may have been due to the blockade of mitochondrial protein synthesis during chronic poisoning, which impairs the repair of damaged cristae [29,30]. Mitochondrial damage can induce apoptosis, causing wrinkling of the nuclear membrane [31]. Additionally, a decrease in antioxidant enzyme levels and increased MDA content were observed, suggesting that AFB_1_ induced oxidative stress in the liver. Altyar et al. [27] found that AFB_1_ significantly increased nitric oxide and MDA levels in the heart and liver of rats while reducing SOD, GPX, GSH, and CAT levels. In addition, previous research in the laboratory has also produced similar findings, showing that AFB_1_ damages the nuclei and mitochondria of liver cells and reduces antioxidant levels [7,22]. These results, in line with our study, indicate that AFB_1_ induces oxidative stress and apoptosis in the liver. This may explain the inhibited growth in the AFB_1_ group.

### 4.4. The Effect of AFB_1_ on Bile Acid Metabolism in Duckling Liver

The liver is the primary site of bile acid synthesis [32]. Bile acids are transported back to the liver via the portal vein after performing their function in the intestine [32]. This study showed that TBA levels in the liver of the AFB_1_ group significantly increased in the second week, with the liver turning yellow-green, indicating abnormal bile metabolism [5,6]. In previous studies, we also found that adding 90 µg/kg of AFB_1_ to the feed can cause bile stasis in the liver of meat ducks in the fourth week [7]. The results of this study are similar to those of previous research. Excess bile in the liver may activate a protective mechanism that reduces bile production, preventing bile acids in the blood from being reused, thus leading to elevated bile acid levels in the serum. Interestingly, the liver did not turn green, nor did bile acid levels show significant changes during the first week. This suggests that the effect of AFB_1_ on bile metabolism may be time-dependent.

### 4.5. Changes in Liver Transcriptome

To date, there have been no reports on the liver transcriptome response after abnormal bile metabolism in ducklings. Transcriptome quality control results in this study showed that all pre-processing steps met sequencing requirements [15,19]. Additionally, the qPCR results closely matched those obtained from transcriptome sequencing, further confirming the reliability of the transcriptome data [15,19]. Differential gene expression analysis classified these genes into four categories: bile acid metabolism, lipid metabolism, oxidative damage, and cancer.

### 4.6. Changes in Bile Acid Synthesis Genes

The liver synthesizes bile acids from cholesterol, with *CYP7A1* acting as the key rate-limiting enzyme [13,32]. *CH24H* and *CH25H* are also critical genes involved in bile acid synthesis [13,33,34]. This study showed that AFB_1_ significantly upregulated these genes, indicating that AFB_1_ increases bile acid synthesis, which may explain the liver’s green color and abnormal bile metabolism. Additionally, *BSEP*, *ABCG5*, and *ABCG8* expression levels in the AFB_1_ group were downregulated. These genes regulate bile acid efflux from the liver, and their reduced expression may hinder this process, leading to increased bile acid content and exacerbating symptoms of bile metabolism abnormalities [17,18,35,36]. Previous reports suggest that AFB_1_ affected the expression of *CYP7A1* and *BSEP* genes in the liver of meat ducks in the fourth week and also affected bile acid metabolism in mice and rats [7,37,38]. This study extends those findings to ducklings, showing that increased bile acid synthesis and impaired efflux contribute to abnormal bile metabolism in ducklings.

### 4.7. Changes in Cholesterol Synthesis Genes

The liver is also a key organ for cholesterol synthesis, with *HMGCS1* and *HMGCR* acting as rate-limiting enzymes [15,16]. This study showed that AFB_1_ significantly downregulated these genes, indicating reduced cholesterol synthesis, which may explain the decrease in serum cholesterol levels. Zhou et al. [39] reported that AFB_1_ increased cholesterol levels, including total cholesterol, in HepG2 liver cancer cells, and Ismail et al. [40] found increased cholesterol levels in rabbit serum. The discrepancy between this study and previous research may be due to dosing differences or ducklings’ heightened sensitivity to AFB_1_ [3]. Further research is needed to clarify these findings.

### 4.8. Changes in Lipid Synthesis Genes

Fatty acid metabolism is another critical liver function [41]. Stearoyl-CoA desaturase (SCD) is a rate-limiting enzyme involved in synthesizing monounsaturated fatty acids, playing a key regulatory role in fatty acid metabolism [42]. This study showed that the expression of *SCD* and *SCD5* was upregulated in the AFB_1_ group. Increased *SCD* expression can lead to triglyceride accumulation, potentially causing fatty liver [42,43]. This may explain the fatty degeneration observed in the AFB_1_ group. Additionally, the expression of *FASN* and *FADS2* was downregulated, possibly due to a negative feedback mechanism triggered by lipid accumulation. Further research is needed to explore this regulation in detail.

### 4.9. Changes in Antioxidant-Related Genes

AFB_1_ can induce the production of ROS, leading to oxidative damage in the liver [3,10,11]. *CAT*, *SOD1*, *GSTK1*, and *MGST2* are genes related to antioxidant activity, and this study showed that AFB_1_ downregulated their expression in the liver [22,44,45]. Wang et al. [22] similarly reported that AFB_1_ significantly reduces the levels of *GPX1*, *SOD1*, and *CAT* in the duckling liver. These results suggest that AFB_1_ impairs the expression of oxidative damage-related genes, contributing to the reduction in antioxidant capacity observed in the AFB_1_ group.

### 4.10. Biological Mechanism of AFB_1_-Induced Poisoning in Ducklings

**Figure 3:**
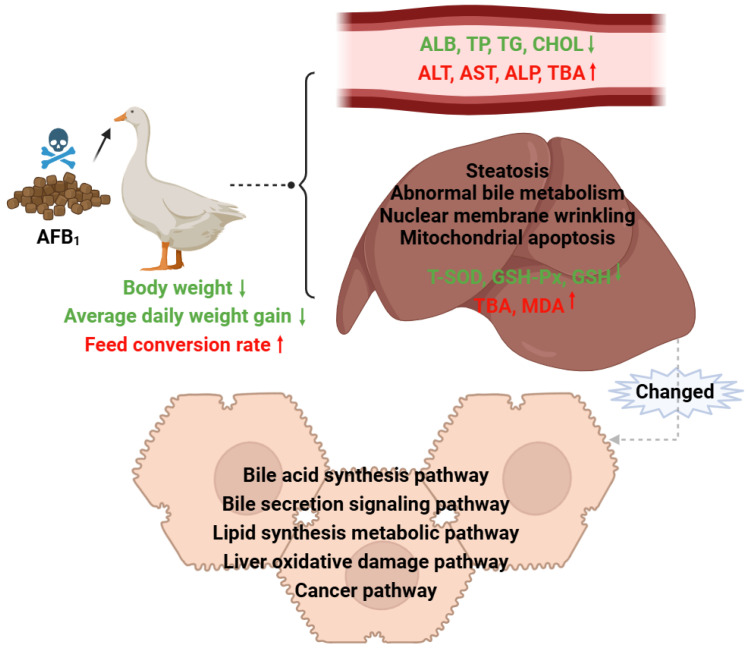
Biological mechanism of AFB_1_-induced abnormal bile metabolism in duckling liver.

AFB_1_ causes changes in the expression levels of genes involved in cholesterol metabolism, lipid metabolism, and antioxidant pathways in the liver. These changes are the main cause of liver damage and subsequently lead to decreased production performance and health impairment in ducklings. Considering the effects of AFB_1_ on the production performance and health of ducklings, as well as the functions of differentially expressed genes from the transcriptome data, a biological mechanism pathway of AFB_1_-induced abnormal bile metabolism in ducklings was developed (Figure 3). AFB_1_ damages liver tissue and organelles, impairing growth. The abnormal expression of related genes in the liver is a key factor contributing to abnormal bile metabolism.

## 5. Conclusions

AFB_1_ not only affects the growth and liver health of ducklings, but it also leads to abnormal bile metabolism in the liver. The abnormal expression of genes related to bile acid metabolism, lipid metabolism, oxidative damage, and cancer may be key factors contributing to AFB_1_-induced liver damage and bile metabolism abnormalities. Additionally, this study was the first to identify a time-dependent effect of AFB_1_ on bile metabolism in ducklings. In summary, this study provides valuable insights for research on bile metabolism abnormalities and the prevention and control of AFB_1_ in poultry farming.

## Figures and Tables

**Table 1:** Nutritional level of basic diet and feed formula ^1^.

Ingredient	Percentage (%)	Nutrient Level	Content
Corn	65.50	ME (kcal/kg)	2800
Soybean meal	30.30	Crude protein (%)	19.00
Soybean oil	1.00	Calcium (%)	0.80
Limestone	1.20	Available phosphorus (%)	0.28
Salt	0.3	Met (%)	0.50
Choline chloride	0.1	Lys (%)	1.12
Lys	0.36	Met + Cys (%)	0.85
Met	0.24		
Premix ^1^	1.00		

^1^ The premix provided the following per kg of diet: vitamin A, 10,000 IU; cholecalciferol, 2500 IU; vitamin E, 35 mg; thiamine, 2.50 mg; riboflavin, 10 mg; pyridoxine, 5.5 mg; iron, 80 mg; manganese, 90 mg; copper, 10 mg; zinc, 60 mg; selenium, 0.4 mg; iodine, 0.3 mg.

**Table 2:** Primers for PCR.

Gene Name	Accession	Primer Sequence (5′-3′)	Product Size
*CYP7A1*	NM_001310351.1	F: CAAAGCAGGAGACCGAGAGA	216 bp
		R: CATTCAGGAACATGCGGAGG	
*CH24H*	XM_027458607.2	F: TGAAAGGAGACGCTCTGGAG	160 bp
		R: TCGGCCTGAACTCTTTCCAT	
*CH25H*	XM_005029425.5	F: CTACGCAATGGACAGACAGC	216 bp
		R: GTTGGATAGTTCTGCGGCTG	
*SLC51B*	XM_005025513.5	F: ATTACTCTGTCCTCGCGCTT	295 bp
		R: ATGGCATCCTCTGAGCTCTC	
*SCD*	XM_027460089.2	F: GGCCATATTCGGGTTGACAC	253 bp
		R: GTCTGCATCTGTCTCGGAGA	
*SCD5*	XM_038177997.1	F: AAGTACATCAACCCCAGGCA	166 bp
		R: CCAACCCCAACCAGAACATG	
*SOD1*	XM_027449207.2	F: TGGACCAAAGGATGCAGAGA	200 bp
		R: CATTCCCAGTTAGCGTGCTC	
*CAT*	XM_027458335.2	F: AATGTGCGTGACTGACAACC	196 bp
		R: ACGTTCATCCTCCTTCAGCA	
*GAPDH*	XM_038180584.1	F: TGAAAGTCGGAGTCAACGGA	249 bp
		R: CACTTGATGTTGCTGGGGTC	

**Table 3:** Effect of AFB_1_ on growth performance and organ weight of ducklings ^1^.

Item	Control	AFB_1_	*p*
Initial weight (g)	50.70 ± 2.15	50.92 ± 2.38	0.87
First week			
Body weight (g)	178.13 ± 13.71 ^a^	161.46 ± 8.27 ^b^	0.03
Average daily weight gain (g)	18.20 ± 2.03 ^a^	15.79 ± 1.01 ^b^	0.03
Average daily feed intake (g)	29.57 ± 2.34	28.19 ± 1.56	0.26
Feed conversion rate	1.63 ± 0.06 ^b^	1.79 ± 0.07 ^a^	<0.01
Liver weight (g)	5.03 ± 0.46	5.32 ± 1.88	0.73
Heart weight (g)	1.15 ± 0.21	1.17 ± 0.33	0.92
Second week			
Body weight (g)	412.92 ± 16.08 ^a^	367.08 ± 23.31 ^b^	<0.01
Average daily weight gain (g)	33.54 ± 0.73 ^a^	28.90 ± 3.45 ^b^	0.01
Average daily feed intake (g)	60.50 ± 3.64	57.50 ± 5.77	0.31
Feed conversion rate	1.80 ± 0.12 ^b^	1.99 ± 0.08 ^a^	0.01
Liver weight (g)	12.67 ± 1.41	11.47 ± 1.66	0.21
Heart weight (g)	3.12 ± 0.28	2.98 ± 0.32	0.46

^1^ The values are expressed as the mean ± standard deviation (*n* = 6). ^a,b^ Means with different superscripts differ significantly (*p* < 0.05); the same applies below.

**Table 4:** Effect of AFB_1_ on serum biochemical indicators and bile acid metabolism indicators ^1^.

Item	Control	AFB_1_	*p*
First week			
ALT (U/L)	34.15 ± 1.98 ^b^	51.05 ± 12.85 ^a^	0.01
AST (U/L)	24.23 ± 8.88 ^b^	45.85 ± 10.53 ^a^	<0.01
ALP (U/L)	852.05 ± 227.78	1114.58 ± 785.40	0.45
TP (g/L)	25.03 ± 3.83 ^a^	17.65 ± 4.27 ^b^	0.01
ALB (g/L)	8.47 ± 1.34 ^a^	5.92 ± 2.26 ^b^	0.04
TG (mmol/L)	2.33 ± 1.02	1.36 ± 0.54	0.07
CHOL (mmol/L)	4.79 ± 1.13	4.66 ± 1.43	0.86
TBA (μmol/L)	36.50 ± 13.94	33.42 ± 18.86	0.75
TBiL (μmol/L)	11.10 ± 4.68	11.90 ± 6.36	0.81
DBiL (μmol/L)	0.35 ± 0.25	0.37 ± 0.24	0.85
Second week			
ALT (U/L)	37.20 ± 6.53 ^b^	55.58 ± 10.88 ^a^	<0.01
AST (U/L)	27.48 ± 15.94 ^b^	52.55 ± 11.15 ^a^	0.01
ALP (U/L)	728.77 ± 108.79 ^b^	1035.43 ± 134.09 ^a^	<0.01
TP (g/L)	31.65 ± 10.34 ^a^	19.52 ± 5.66 ^b^	0.03
ALB (g/L)	8.85 ± 1.86 ^a^	5.90 ± 2.19 ^b^	0.03
TG (mmol/L)	1.23 ± 0.28 ^a^	0.50 ± 0.19 ^b^	<0.01
CHOL (mmol/L)	5.02 ± 1.15 ^a^	3.70 ± 0.88 ^b^	0.04
TBA (μmol/L)	13.15 ± 4.04 ^b^	26.97 ± 5.82 ^a^	<0.01
TBiL (μmol/L)	6.68 ± 2.05	12.00 ± 6.65	0.09
DBiL (μmol/L)	0.40 ± 0.12	0.63 ± 0.48	0.27

^1^ Values are expressed as means ± SD (*n* = 6), and different superscripts in different columns of the same row indicate significant differences (*p* < 0.05).

**Table 5:** Effect of AFB_1_ on liver bile acid and liver antioxidant enzyme content ^1^.

Item	Control	AFB_1_	*p*
First week			
TBA (μmol/L)	14.25 ± 1.80	14.70 ± 3.93	0.80
T-SOD (U/mg protein)	182.53 ± 11.63 ^a^	140.96 ± 21.73 ^b^	<0.01
MDA (nmol/mg protein)	1.68 ± 0.21	1.96 ± 0.33	0.11
GSH-Px (U/mg protein)	89.25 ± 8.53 ^a^	67.23 ± 8.86 ^b^	<0.01
GSH (U/mg protein)	46.75 ± 5.78 ^a^	38.48 ± 4.19 ^b^	0.02
Second week			
TBA (μmol/L)	15.84 ± 3.66 ^b^	23.61 ± 3.59 ^a^	<0.01
T-SOD (U/mg protein)	177.71 ± 24.95 ^a^	137.97 ± 15.58 ^b^	<0.01
MDA (nmol/mg protein)	1.59 ± 0.20 ^b^	2.01 ± 0.16 ^a^	<0.01
GSH-Px (U/mg protein)	87.13 ± 11.67 ^a^	64.38 ± 6.78 ^b^	<0.01
GSH (U/mg protein)	45.26 ± 5.78 ^a^	36.53 ± 3.37 ^b^	0.01

^1^ Values are expressed as means ± SD (*n* = 6), and different superscripts in different columns of the same row indicate significant differences (*p* < 0.05).

**Table 6:** Statistical summary of the liver RNA sequencing datasets.

Sample	Raw Reads	Q30(%) Value ^1^	Clean Reads	Total Mapped
Control-1	43,696,008	96.29	43,407,312	37,874,240 (87.25%)
Control-2	42,541,812	95.70	42,087,594	32,070,452 (76.20%)
Control-3	41,682,592	95.76	41,254,000	32,749,563 (79.39%)
Control-4	44,565,508	95.58	44,055,108	34,348,062 (77.97%)
Control-5	37,918,248	95.69	37,557,418	29,587,206 (78.78%)
Control-6	40,055,144	95.55	39,694,084	31,527,638 (79.43%)
AFB_1_-1	42,201,282	95.42	41,738,988	31,248,957 (74.87%)
AFB_1_-2	43,191,696	95.63	42,735,964	32,396,410 (75.81%)
AFB_1_-3	42,490,296	95.55	42,111,914	31,652,071 (75.16%)
AFB_1_-4	39,233,296	95.50	38,850,434	29,469,930 (75.85%)
AFB_1_-5	39,513,334	95.54	39,141,620	30,590,468 (78.15%)
AFB_1_-6	39,724,756	95.44	39,293,828	29,774,682 (75.77%)

^1^ Q30 value refers to the sequencing quality score, indicating a 0.1% probability of error.

**Table 7:** Changes in differential gene expression levels in various metabolic pathways of duckling liver.

Gene ID	Gene Symbol	Log2 (Fold Change)	Gene Description
Bile acid synthesis pathway			
16440	*HSD17B4*	−1.16	hydroxysteroid 17-beta dehydrogenase 4
19755	*CH25H*	1.43	cholesterol 25-hydroxylase
3365	*CYP7A1*	2.18	cholesterol 7-alpha-monooxygenase
1859	*HSD3B7*	−0.92	hydroxy-delta-5-steroid dehydrogenase, 3 beta- and steroid delta-isomerase 7
20691	*LOC101805427*	−1.13	24-hydroxycholesterol 7-alpha-hydroxylase
12643	*LOC101797844*	0.60	acyl-coenzyme A amino acid N-acyltransferase 2
16937	*AMACR*	−1.21	alpha-methylacyl-CoA racemase
17396	*ACOX2*	−1.00	acyl-CoA oxidase 2
679	*LOC101791081*	−1.98	25-hydroxycholesterol 7-alpha-hydroxylase
17705	*SCP2*	−0.89	sterol carrier protein 2
11512	*CH24H*	0.95	cholesterol 24-hydroxylase
11839	*LOC101802865*	−1.47	sterol 26-hydroxylase, mitochondrial
Bile secretion signaling pathway			
9766	*BSEP*	−1.05	ATP binding cassette subfamily B member 11
12806	*ATP1B4*	−1.45	ATPase Na+/K+ transporting family member beta 4
2095	*ATP1B3*	0.95	ATPase Na+/K+ transporting subunit beta 3
8315	*NCEH1*	1.27	neutral cholesterol ester hydrolase 1
16116	*SLC51B*	−0.53	solute carrier family 51 beta subunit
11264	*ABCB5*	1.73	ATP binding cassette subfamily B member 5
15489	*ADCY2*	−1.67	adenylate cyclase 2
17247	*SLC51A*	0.81	solute carrier family 51 alpha subunit
14631	*ADCY3*	2.20	adenylate cyclase 3
14939	*KCNN2*	−0.65	potassium calcium-activated channel subfamily N member 2
19874	*ATP1B1*	−1.13	ATPase Na+/K+ transporting subunit beta 1
6195	*ABCG8*	−3.41	ATP binding cassette subfamily G member 8
6196	*ABCG5*	−4.00	ATP binding cassette subfamily G member 5
11334	*SCTR*	3.24	secretin receptor, transcript variant X1
15107	*NR1H4*	−0.52	nuclear receptor subfamily 1 group H member 4
Lipid synthesis metabolic pathway			
6139	*ACSL4*	1.02	acyl-CoA synthetase long-chain family member 4
12495	*ACADL*	−0.34	acyl-CoA dehydrogenase, long chain
14836	*FADS2*	−0.79	fatty acid desaturase 2
15821	*SCD*	2.34	stearoyl-CoA desaturase
7095	*ACOX3*	−1.12	acyl-CoA oxidase 3
5424	*ACSL5*	−1.46	acyl-CoA synthetase long-chain family member 5
6369	*FABP3*	2.87	fatty acid binding protein 3
7566	*CD36*	−1.63	CD36 molecule, transcript variant X6
1228	*PPARA*	−0.94	peroxisome proliferator activated receptor alpha
4591	*SCD5*	1.72	stearoyl-CoA desaturase 5
17466	*HMGCS1*	−0.81	3-hydroxy-3-methylglutaryl-CoA synthase 1
2844	*HMGCR*	−0.56	3-hydroxy-3-methylglutaryl-CoA reductase
2806	*FASN*	−1.81	fatty acid synthase
17756	*ACAA1*	−0.47	acetyl-CoA acyltransferase 1
Liver oxidative damage pathway			
13087	*MGST2*	−0.74	microsomal glutathione S-transferase 2
7302	*GSTK1*	−1.01	glutathione S-transferase kappa 1
14244	*GGT1*	1.31	gamma-glutamyltransferase 1
1745	*GPX3*	1.26	glutathione peroxidase 3
386	*GCLC*	1.32	glutamate-cysteine ligase catalytic subunit
392	*LOC101797138*	3.32	glutathione S-transferase, transcript variant X2
394	*LOC101798048*	0.77	glutathione S-transferase, transcript variant X1
19681	*GPX4*	1.20	glutathione peroxidase 4
17700	*GPX7*	2.19	glutathione peroxidase 7
10599	*GPX8*	0.97	glutathione peroxidase 8
8664	*SOD1*	−0.36	superoxide dismutase 1
2543	*CAT*	−1.38	catalase
9868	*PXMP4*	−1.25	peroxisomal membrane protein 4
19397	*PEX6*	−0.42	peroxisomal biogenesis factor 6
8522	*PEX7*	−0.80	peroxisomal biogenesis factor 7
Cancer pathway			
9473	*MAP2K2*	−0.48	mitogen-activated protein kinase kinase 2
5570	*LRP5L*	−1.27	LDL receptor related protein 5 like
13087	*MGST2*	−0.74	microsomal glutathione S-transferase 2
1418	*WNT5B*	1.41	Wnt family member 5B
13470	*CCND1*	1.29	cyclin D1
6331	*PIK3CD*	−2.21	phosphatidylinositol-4,5-bisphosphate 3-kinase catalytic subunit delta
9391	*PRKCB*	−0.44	protein kinase C beta
14575	*CDKN1A*	2.95	cyclin dependent kinase inhibitor 1A
8778	*AKT3*	0.79	AKT serine/threonine kinase 3
11885	*FGFR2*	1.00	fibroblast growth factor receptor 2
13263	*IGF1*	−1.53	insulin like growth factor 1
16734	*IGF2*	−0.55	insulin like growth factor 2
6458	*FGF2*	1.18	fibroblast growth factor 2
12322	*PTK2*	−0.77	protein tyrosine kinase 2

## Data Availability

The data presented in this study are available on request from the corresponding author (the data are not publicly available due to privacy or ethical restrictions).

## Supplementary Materials

The following supporting information can be downloaded at https://www.mdpi.com/article/10.3390/ani14202996/s1: Supplementary Document S1. Differential Gene Expression Details Table.
